# Physiological characterization of chitin synthase A responsible for the biosynthesis of cuticle chitin in *Culex pipiens pallens* (Diptera: Culicidae)

**DOI:** 10.1186/s13071-021-04741-2

**Published:** 2021-05-01

**Authors:** Xiaoshan Yang, Yang Xu, Qi Yin, Hongbo Zhang, Haitao Yin, Yan Sun, Lei Ma, Dan Zhou, Bo Shen

**Affiliations:** 1 Department of Pathogen Biology, Nanjing Medical University, Nanjing, China; 2 Experimental Teaching Center of Basic Medicine, Nanjing University of Chinese Medicine, Nanjing, China

**Keywords:** Chitin, Chitin synthase, Molting, Mosquito, Insect

## Abstract

**Background:**

The pathogens transmitted by mosquitoes to humans and animals cause several emerging and resurgent infectious diseases. Increasing insecticide resistance requires rational action to control the target vector population. Chitin is indispensable for insect growth and development and absent from vertebrates and higher plants. Chitin synthase A (CHSA) is a crucial enzyme in chitin synthesis; therefore, identifying and characterizing how CHSA determines chitin content may contribute to the development of novel vector control strategies.

**Results:**

The injection of small interfering RNA targeting *CHSA* (siCHSA) to knockdown *CHSA* transcripts in larval, pupal and adult stages of *Culex pipiens pallens* resulted in the appearance of different lethal phenotypes. When larval and pupal stages were injected with siCHSA, *CHSA* knockdown prevented larval molting, pupation and adult eclosion, and affected the production of chitin and chitin degradation, which resulted in an ecdysis defect phenotype of mosquitoes. When siCHSA was injected into mosquitoes in the adult stage, *CHSA* knockdown also affected the laminar organization of the mesoderm and the formation of pseudo-orthogonal patterns of the large fibers of the endoderm.

**Conclusion:**

We provide a systematic and comprehensive description of the effects of *CHSA* on morphogenesis and metamorphosis. The results show that CHSA not only affects chitin synthesis during molting, but also might be involved in chitin degradation. Our results further show that CHSA is important for the structural integrity of the adult mosquito cuticle.

**Graphic abstract:**

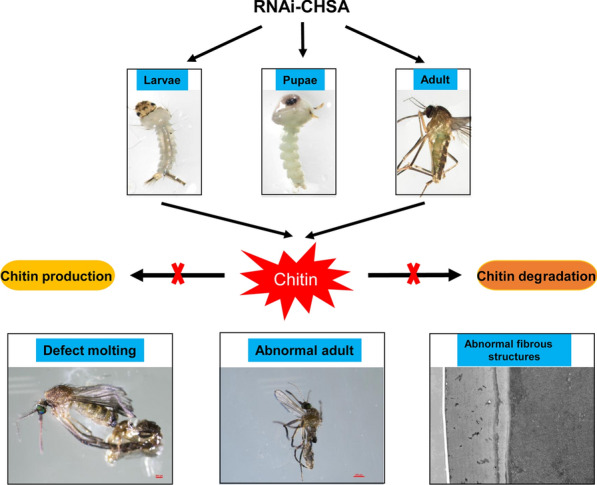

**Supplementary Information:**

The online version contains supplementary material available at 10.1186/s13071-021-04741-2.

## Background

Mosquitoes are the most dangerous animals in the world, causing hundreds of thousands of deaths every year by spreading arboviruses and parasites [1–5]. Currently, chemical insecticides are the main agents used to control insect vectors and reduce disease transmission. However, the widespread use of chemical control has led to the development of insect resistance, the resurgence of target pests, food and environment pollution and the destruction of non-target insects [6–8]. The environmental and health impacts of the toxicity of conventional insecticides have become increasingly unacceptable [9]. Therefore, there is an urgent need to develop novel, safer bioinsecticides with low toxicity [10]. The normal growth and development of insects is inseparable from the process of molting [11], and many researchers have investigated growth metabolic regulation and metabolism strategies in insects to identify novel targets for insect control [12–17].

Molting is a crucial process in insect growth metabolism. The process of insect molting involves the biosynthesis, transformation and modification of chitin [18]. Chitin, a linear polysaccharide of the amino sugar *N*-acetyl glucosamine, is the main component of insects’ extracellular barrier, such as the cuticle and the peritrophic matrix (PM). Chitin is also an important component of the tracheal system, reproductive ducts and the ducts of various dermal glands in the internal structures of many insects [6, 9]. It plays an important role in supporting muscle attachment for movement, preventing chemical and physical damage and preventing infectious diseases, thereby representing the first line of defense in challenging environments [19, 20].

The chitin biosynthesis pathway involves eight key regulatory enzymes. The last step is executed by the chitin synthases (CHSs; UDP-*N*-acetyl-d-glucosamine: chitin 4-beta-*N*-acetylglucosaminyltransferase), which form a large group of plasma membrane proteins belonging to family 2 of the glycosyltransferase subclass of enzymes [21]. Chitin biosynthesis plays an irreplaceable role in the growth of insects, requiring modification and physiological regulation at developmental stages. Chitin is completely absent from vertebrates and higher plants [22, 23] and thus has generated interest as a potential target for ecologically friendly insecticides; therefore, an understanding of chitin biosynthetic pathways could provide additional strategies for pest control. Chitin synthesis inhibitors can prevent insects from molting by interfering with chitin biosynthesis [8]. Among these, the benzylphenolurea (BPU) insecticides have shown great potential to inhibit chitin biosynthesis; however, the non-target effects of BPUs can adversely affect beneficial species, such as bees, making them a controversial group of insecticides [7]. Therefore, it is necessary to study CHS (EC 2.4.2.16) to find new and effective target sites to combat insect pests.

Insects commonly possess two chitin synthase genes. Class A CHSs (CHSAs) are primarily involved in chitin synthesis for the exoskeleton cuticle [24–28]; class B CHSs (CHSBs) play a major role in the synthesis of chitin in the intestinal PM [9, 29]. Currently available data on biochemically characterized insect CHSs show that insect CHSA is associated with insect molting [30, 31] and is a crucial enzyme that balances growth and development. RNA interference (RNAi) refers to use of double-stranded RNA (dsRNA) or small interfering RNA (siRNA) to silence a transcribed gene, block the mRNA of the gene or convert it into active gene products. Recent studies have demonstrated that the use of RNAi technologies in insect management programs has great potential to control agriculturally important insect pests [32]. In* Manduca sexta* [33] and* Spodopetera exigua* [34], the application of RNAi to silence CHSA resulted in disruption of the cuticular exoskeleton and tracheal ectodermis. In* Tribolium castaneum* and* Locusta migratoria manilensis*, CHSA was found to be required for larval–larval processes, and for larval–pupal and pupal–adult processes [35]. In* Tribolium castaneum*, CHSA plays a prominent role in embryo development and oviposition [31]. In* Anopheles gambiae*, CHSA was detected in newly formed compound eyes [26]. In* Aedes aegypti*, dsRNA applied* via* direct addition to the breeding water reduced the amount of CHSA and B transcripts [6]. However, those studies were performed in different developmental stages of different species, and the mechanism by which CHSA affects the molting process in mosquitoes remains unclear. Studying CHSA function in mosquitoes is crucial to acquire a full understanding of the regulatory processes of mosquito growth and reproduction, and may lead to the utilization of the CHSA gene in new approaches to insect control.

*Culex pipiens pallens* is a common house mosquito and is a vector of West Nile virus (WNV), epidemic encephalitis, *Wucheraria bancrofti* and *Brugia malavi* [36]. In the present study, our aim was to identify and characterize the *CHSA* gene from *C. pipiens pallens* (*CpCHSA*). We used RNAi to identify whether CpCHSA is essential for growth and development at different development stages and in different tissues of *C. pipiens pallens*. The results indicated that CpCHSA affects chitin synthesis and degradation, which is necessary for molting processes. CpCHSA is also important for cuticle formation in adult stages, playing critical roles in endocuticle development.

## Methods

### Mosquito rearing

The laboratory strain of *C. pipiens pallens* originated from a sample collected from a field in Tangkou County of Shandong Province in 2009 that was then transported to our laboratory. *Culex pipiens pallens* were reared at 28 ± 1 °C and 70–80% relative humidity under a 12/12-h light/dark photoperiod. The larvae were fed rat chow. Adult mosquitoes were maintained on 7% sucrose solution. The female mosquitoes were fed fresh mouse blood to induce egg-laying. Mosquitoes were not treated with insecticides or other chemicals [37].

### *CpCHSA* cDNA cloning and analysis

RNA was isolated using RNAiso Plus reagent (Takara, Tokyo, Japan). The full-length sequence of *CpCHSA* complementary DNA (cDNA) was determined from seven overlapping PCR fragments (Additional file 1: Table S1). The 5′- and 3′-end fragments were obtained using rapid amplification of cDNA ends (RACE) using a SMARTer RACE 5′/3′ Kit (Takara). PCR amplification products were analyzed using agarose gels and then purified (TIANGEN, Beijing, China). Purified DNA was ligated into vector pClone007 (TSINGKE, Nanjing, China) and sequenced. The obtained full-length cDNA of *CpCHS*A was submitted to the National Center for Biotechnology Information (NCBI) and assigned accession number MH013352.

The conceptual translation of the amino acid sequence of CpCHSA was performed using the ExPASy website (https://web.expasy.org/translate/). The molecular weight, isoelectric point, transmembrane helices and *N*-glycosylation sites were predicted using the websites (http://cn.expasy.org/tools/pi_tool.html) and (http://www.cbs.dtu.dk/services/NetNGlyc/). The amino acid sequence of the conserved catalytic domain was predicted based on a previous study [12].

### Analysis of gene expression patterns

Total RNA was isolated from the eggs (at 0 and 24 h), instar larvae (first, second, third [L3] and fourth [L4] instar larvae), pupal stage (0 and 24 h) and adult stage (1–3 days post-eclosion [PE] and 1–3 days post-blood meal [PBM]) to investigate the *C. pipiens pallens* developmental expression profile. The head, foregut, midgut, hindgut, Malpighian tubules, legs, wings, ovaries and carcass were dissected from fourth-instar larvae or 3-day-old adult mosquitoes for tissue-specific expression analysis. Total RNA was isolated from the whole bodies of five mosquitoes and the tissues of ten mosquitos for biological replicates. First-strand cDNA was synthesized using PrimeScript RT Master Mix (Takara). A LightCycler® 96 Instrument was used for quantitative real-time PCR (qPCR) analysis (Roche, Basel, Switzerland) with the BrightGreen 2× qPCR MasterMix-No Dye (Applied Biological Materials, Vancouver, BC, Canada) and the cDNA as the template. Specific primers are shown in Additional file 1: Table S2. The qPCR reaction volume (10 μl) contained the Power SYBR Green PCR Master Mix, specific primer sequences and diluted cDNA (1 mg/ml). The relative expression levels were normalized to the internal control *ACTB* (encoding β-actin) using the 2^−ΔΔCt^ method [38]. All experiments were performed with three biological replicates. For the analysis of gene expression patterns, the relative expression data were based on the lowest expression value.

### Microinjection

RNA interference was used to knockdown the expression of *CpCHSA*. The siRNA sequences used to silence the *CpCHSA* gene (siCHSA) and negative control (NC) are shown in Additional file 1: Table S3; both were designed and manufactured by Gene Pharma (Shanghai, China). siCHSA or NC (0.30 μg) was injected into the abdomen of L3 and thorax of adult female mosquitoes (12–24 h after PE); in the pupal stage (0–1 h after pupation), they were injected into the dorsal cuticle between the thorax and the abdomen. Both the wild-type (WT) group and NC group served as controls. Total RNA was isolated from whole mosquitoes (*n* ≥ 5), and *CpCHSA* transcript levels were analyzed after RNAi using qPCR. In the L3 and adult stage, the interference efficiency was detected 72 h after RNAi of *CpCHSA.* In the pupal stage, the interference efficiency was detected 24 h after RNAi of *CpCHSA.*

### Analysis of survival

Fifty mosquitoes were injected with siCHSA, and after 24 h, the dead mosquitoes were removed. The remaining mosquitoes were recorded as the number of effective subjects. The number of dead mosquitoes was counted once a day, and L4 and pupae were distinguished according to their body length and morphology under the microscope; the mortality rate was calculated according to the number of dead larvae and pupae, respectively. The pupae that failed to separate from the old cuticle within 24 h of molting were counted as dead; the adults that succeeded in separating from the old cuticle but could not fly for food were counted as a molting death if they died within 1–7 days [26]. For survival analysis, we used the Kaplan–Meier method.

### Chitin staining

The chitin content of the abdominal integuments was quantified after siCHSA or NC injection using chitin staining. Fifty pupae (0–1 h) were randomly selected to be injected with siRNA, and at 12 and 24 h after injection the pupal abdomen was dissected. Slides of paraffin-embedded tissue were deparaffinized in xylene and rehydrated using an ethanol gradient. Fluorescent Brightener 28 (Sigma-Aldrich, Hamburg, Germany) was used to stain the sample, propidium iodide was used as a counterstain, anti-fluorescent stain was added and the samples were then observed under an AXIO confocal fluorescence microscope (Carl Zeiss AG, Oberkochen, Germany) [39].

### Immunofluorescence

For the immunofluorescence experiment, rabbit polyclonal antibodies were prepared against CpCHSA. We designed the peptide antigen of CpCHSA by analyzing the cDNA and protein sequences (Additional file 1: Table S4). The peptide was synthesized and then subcloned into pET-28A-SUMO and PGEX-4T-AB1 transfer plasmids. ABclonal Biotechnology (Wuhan, China) synthesized the gene and produced the polyclonal antibodies. The acquired antibodies were tested to ensure that they met the experimental requirements.

To analyze the localization of the CpCHSA protein, we selected 50 pupae (0–1 h) to be injected with siCHSA, and then collected pupa samples at 12 and 24 h after injection. Paraffin sections were made from pupae treated with siCHSA or NC. The tissues were fixed in 4% paraformaldehyde at 4 °C overnight, then sectioned. The sections then deparaffinized in two washes of xylene (each wash 15 min), rehydrated through successive baths of ethanol (100, 96 and 70% in water, 15 min each), washed twice for 5 min and then once with PBST (0.01 M phosphate-buffered saline [PBS], pH 7.4 containing 0.1% Tween 20) for 10 min. The sections were blocked with 2% bovine serum albumin for 30 min, followed by incubation with anti-CpCHSA antibodies (1:100) at 4 °C overnight. The sections were then washed and then incubated with Alexa Fluor® 594-conjugated donkey anti-rabbit IgG (Abcam, Cambridge, UK) secondary antibody (1:200 in blocking buffer) for 50 min, in the dark. After three washes in PBST for 5 min each, the nuclei were stained with 4′,6-diamidino-2-phenylindole (DAPI) for 10 min in the dark. The sections were then washed with PBST and observed under a fluorescence microscope (Carl Zeiss AG) [26], The fluorescence was analyzed using Image J software (National Institutes of Health, Bethesda, MD, USA).

### Electron microscopy

Third-instar larvae injected with siCHSA or NC were collected in adult mosquitoes at 1 day PE; pupae (0–1 h) injected with siCHSA or NC were collected in the pupal stage at 12 and 24 h after injection and in adult mosquites at 1 day PE; and adults (12–24 h PE) injected with siCHSA or NC were collected at 72 h after injection. Samples were collected and fixed in 4% paraformaldehyde at 4 °C, and then washed with three times in PBS three times (each wash 15 min). The samples were postfixed in 1% OsO_4_ in 0.1 M PBS (pH 7.4) for 2 h at room temperature, following which the OsO_4_ was removed and the samples were rinsed three times in PBS (0.1 M, pH 7.4) (each wash 15 min). The samples were then dehydrated through successive concentrations of ethanol (50, 70, 80, 90, 95 and 100%, for 15 min) and finally through two changes of acetone for 15 min before being infiltrated consecutively with 1:1 acetone:EMBed 812 for 3 h, 2:1 acetone:EMBed 812 overnight and pure EMBed 812 for 7 h. The samples were kept at 37 °C overnight and then baked at 60 °C for 48 h. Sections were cut and stained with uranyl acetate for 15 min followed by lead citrate staining for 15 min. The sections were then air dried overnight. The ultrastructure of the cuticles was then analyzed using transmission electron microscopy (TEM) [40].

### Western blotting

Western blotting was used to evaluate the specificity of the anti-CpCHSA antiserum and to verify the knockdown efficiency of RNAi. L3, pupae (0–1 h) and adults (12–24 h PE) were injected with siCHSA or NC, respectively. Total proteins were extracted from the whole body of larvae (72 h after injection), pupae (24 h after injection) or adult mosquitoes (3 days PE) using radioimmunoprecipitation assay (RIPA) buffer containing 1 mM phenylmethylsulfonyl fluoride (PMSF) and a protease inhibitor cocktail (Thermo Fisher Scientific, Rockford, IL, USA) and then centrifuged at 12,000 *g* at 4 °C for 30 min. A bicinchoninic acid (BCA) protein assay kit (Beyotime, Shanghai, China) was used for protein quantification. The proteins were fractionated using 5% sodium dodecyl sulfate polyacrylamide gel electrophoresis (SDS-PAGE) and transferred to nitrocellulose membranes. The membranes were probed using anti-CpCHSA (1:1000) and β-actin (1:7000; ABclonal Biotechnology) antibodies, followed by incubation with labeled secondary antibodies and visualization of the immunoreactive protein bands. The bands were analyzed using Image J software [41].

### Statistical analysis

Statistical analyses were performed using SPSS version 23.0 (IBM Corp., Armonk, NY, USA) and GraphPad Prism 6.0 software (GraphPad Software Inc., La Jolla, CA, USA) [39]. Student’s t-test or one-way analysis of variance (ANOVA) was used to compare the difference between treatments. The statistical significance of the RNAi knockdown efficiency and the survival rate were analyzed using the unpaired Student’s t-test. To analyze expression levels, we used ANOVA followed by Tukey’s post-hoc test. For survival analysis, we used the Kaplan–Meier curve [42]. Asterisks indicate the statistical significance: *p < 0.05, **p < 0.01, and ***p < 0.001. All experiments were performed using at least three independent cohorts.

## Results

### *CpCHSA* cDNA

The *CpCHSA* cDNA was isolated using RACE-PCR to amplify the 5′ and 3′ regions (Additional file 2: Figure S1). The full-length *CpCHSA* cDNA (GenBank ID: MH013352) comprises 5396 nucleotides, of which 4740 nucleotides encode a putative protein of 1579 amino acid residues with a calculated molecular mass of about 179.54 KDa. The signature motifs “QRRRW” and “EDR” for chitin synthases, which contribute to catalysis and substrate binding, were also present in *CPCHSA* [43]. The CpCHSA protein contains three predicted domains; an N-terminal domain with seven transmembrane helices, a highly conserved central domain and a C-terminal domain with an additional seven transmembrane helices [12].

### *CpCHSA* gene expression pattern

**Fig. 1:**
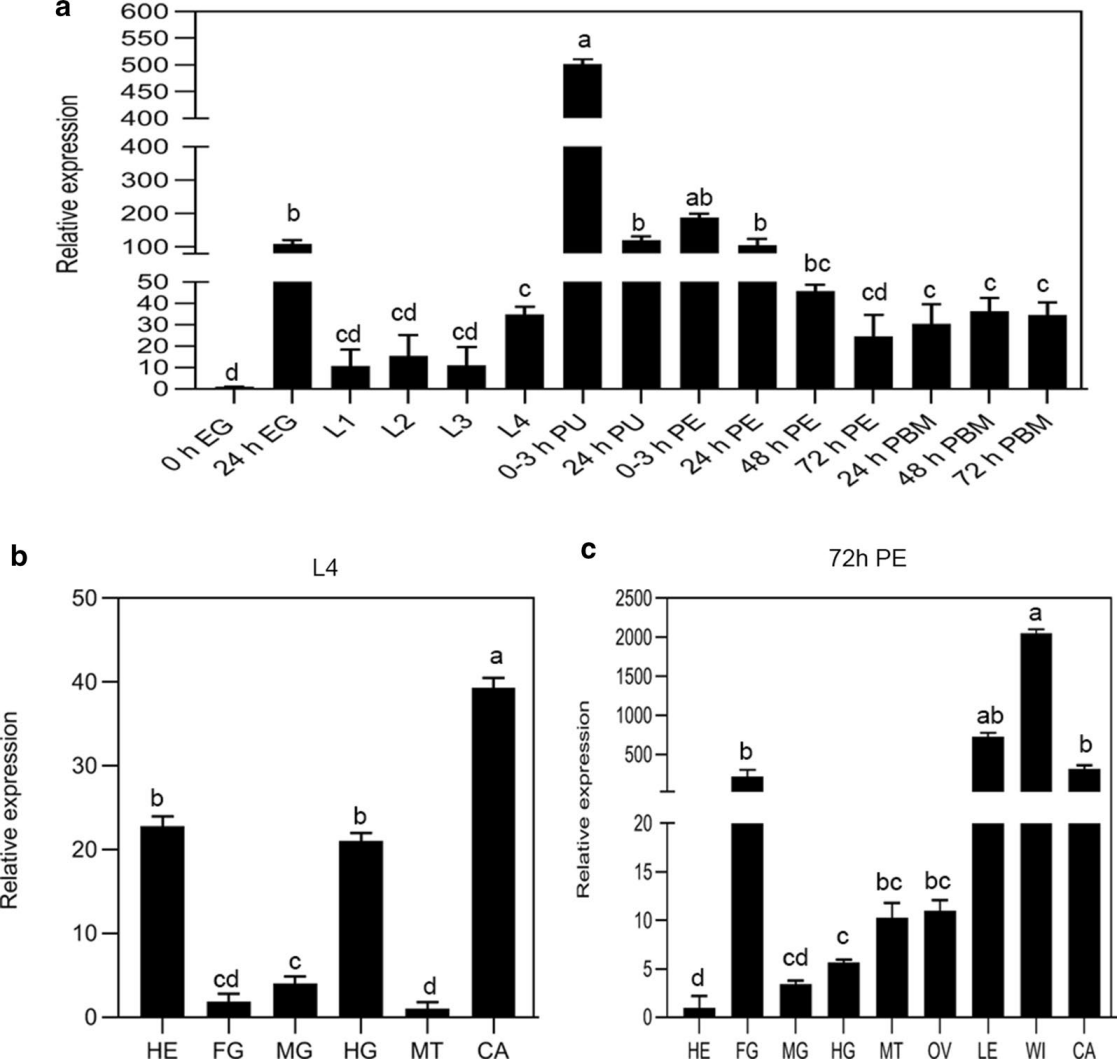
*CpCHSA* gene expression patterns. Relative expression levels of *CpCHSA* in different stages and different tissues, as assessed using quantitative real-time PCR (qPCR). **a** Relative expression at different stages: egg (*EG*), larvae (*L1*–*4*), pupae (*PU*), adult post-eclosion (*PE*) and adult post-blood meal (*PBM*). Five mosquitoes were collected from each group for RNA extraction. Relative expression levels were calculated in comparison with that at 0 h EG, which was ascribed an arbitrary value of 1.** b**,** c** Relative expression level of *CpCHSA* in L4 (**b**) and adult mosquito at 72 h PE (**c**). Tissues included the head (*HE*), foregut (*FG*), midgut (*MG*), hindgut (*HG*), Malpighian tubules (*MT*), ovary (*OV*), leg (*LE*), wing (*WI*) and carcass (*CA*). Tissues from ten mosquitoes were collected from each group for RNA extraction. Relative expression levels were calculated in comparison with expression in MT (**b**) and HE (**c**), which were ascribed an arbitrary value of 1. The *ACTB* (β-actin) gene was used as an internal reference. Data are from three independent experiments with three biological replicates. Different lowercase letters above the bars indicate a statistically significant difference at *P* < 0.05 (analysis of variance [ANOVA]); the same letter indicates data are not significantly different. *CpCHSA* Chitin synthase A gene (CHSA) from *Culex pipiens pallens*.* L1*,* L2*,* L3*,* L4* First-, second-, third-, fourth-instar larvae, respectively

To confirm the role of *CpCHSA* in *C. pipiens pallens*, we first examined the mRNA expression of *CpCHSA* in whole eggs (at 0 and 12 h), larval instars (first, second, third and fourth), pupae (at 0 and 24 h), adults (1–3 days PE) and adults (1–3 days) PBM. The qPCR results indicated that *CpCHSA* is expressed in all of these different developmental stages, with predominant expression in the eggs, pupae and adult (Fig. 1a). We also examined the tissue-specific expression patterns of *CpCHSA*. *CpCHSA* transcripts were significantly enriched in the head, carcass and hindgut of the L4 (Fig. 1b); and in the foregut, leg, wing, and carcass of 3-day PE female mosquitoes (Fig. 1c).

### RNAi of *CpCHSA* hinders molting of *C. pipiens pallens*

**Fig. 2:**
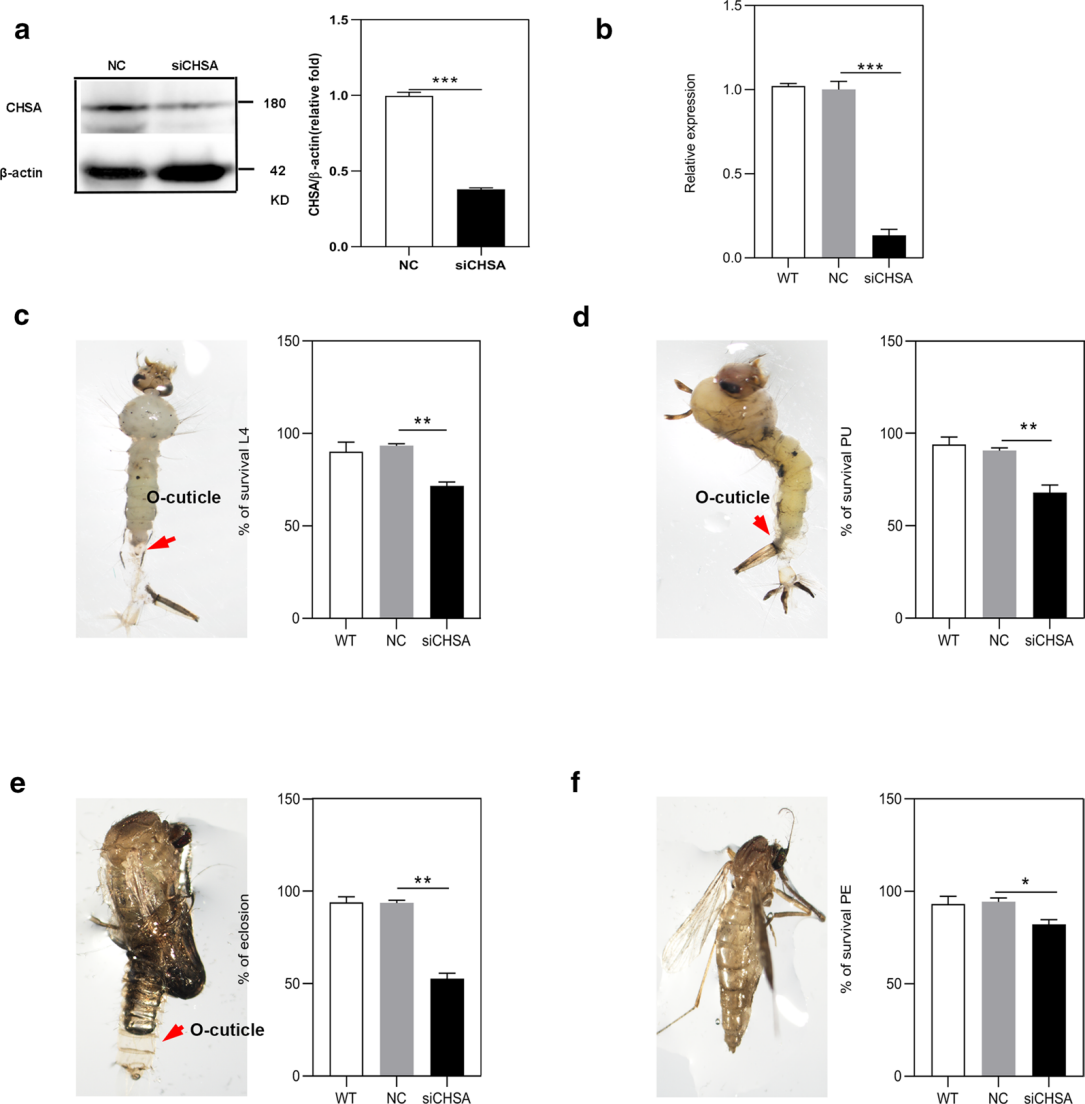
Phenotypes produced by RNA interference (RNAi) of *CpCHSA* in the larvae stage (*n* = 150). The red arrow (**c**,** d**) indicates the shedding of old cuticle (*O-cuticle*) in the defective molting process. **a**,** b** RNAi of *CpCHSA* in L3. Expression levels of *CpCHSA* at 72 h after injection of siCHSA, as assessed using western blotting (**a**) and qPCR (**b**). Five mosquitoes were collected from each group for protein or RNA extraction. **c** siCHSA injection into L3 reduce the survival rate of L4. **d** The survival rate of L4 to the pupal stage. **e** The eclosion rate of pupae. **f** The survival rate of PE mosquitoes. Data represent three biological replicates (50 individuals in each replicate) with three technical replicates, and the results are shown as the mean ± standard error of the mean (SEM). Asterisks indicate a significant difference at *P < 0.05, **P < 0.01, ***P < 0.001, according to Student’s t-test. *NC* Negative control,* siCHSA* short interfering RNA sequences used to silence the *CpCHSA* gene,* WT* wild type

**Fig. 3:**
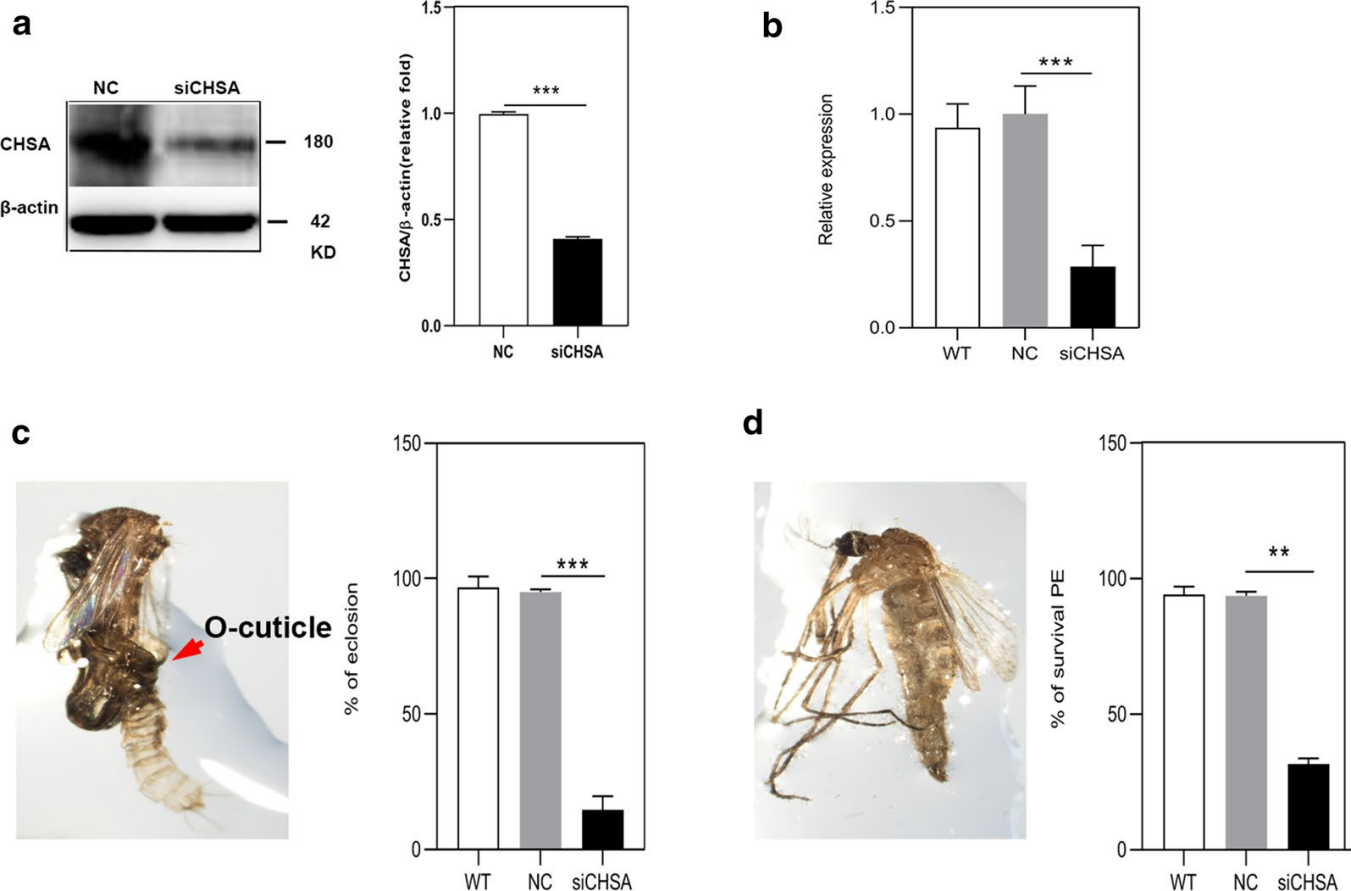
Phenotypes produced by RNAi of *CpCHSA* in the pupal stage (*n* ≥ 50). siCHSA treatment in the 0- to 1-h pupae. **a** CpCHSA protein levels were determined using western blotting analysis with CpCHSA-specific polyclonal antibodies at 24 h after injection of siCHSA. **b** qPCR analysis of *CpCHSA* gene expression at 24 h after the injection of siCHSA. **c** siCHSA injection into pupae reduced the eclosion rate. **d** The survival rate of PE mosquitoes. All surviving individuals were used for measurement. Data represent three biological replicates (50 individuals in each replicate) with three technical replicates, and the results are shown as the mean ± SEM. Asterisks indicate a significant difference at *P < 0.05, **P < 0.01, ***P < 0.001, according to Student’s t-test

To investigate the impact of *CpCHSA* on the molting process, we first monitored molting after knockdown of *CpCHSA* in L3 (*n* = 50) and pupae (*n* = 50). The larvae (Fig. 2) and pupae (Fig. 3) injected with siCHSA displayed a molting defect phenotype at the third to fourth instar, the fourth instar to pupal stage and the pupal stage to adult stage. Specifically, the old cuticle was incompletely separated from the mosquito body. Adult mosquitoes exhibited prominent deformities of the leg, abdomen and wings. *CpCHSA*-deficient adults had difficulty righting themselves and taking off compared with control adults.

In the L3 (Fig. 2), RNAi of *CpCHSA* led to a substantial decrease of 62.9% after 72 h (*p* < 0.0001, unpaired Student’s t-test) in the CpCHSA protein level (Fig. 2a) and a decrease of 86.7% (*P* < 0.0001 unpaired Student’s t-test) in the expression of the *CpCHSA* mRNA (Fig. 2b). Among the 50 larvae injected with siCHSA, 23.5% (*p* = 0.0035, unpaired Student’s t-test) died during the molting process from the third instar to the fourth stage (Fig. 2c), 31.71% (*P* = 0.0071, unpaired Student’s t-test) of the survivors died during the molting process from the fourth instar to the pupal stage (Fig. 2d), 48.7% (*p* = 0.00127, unpaired Student’s t-test) of the survivors died during the molting process from pupal to adult (Fig. 2e) and 20.1% of the survivors (*P* = 0.00127, unpaired Student’s t-test) died during the PE stage (Fig. 2f). In the pupal stage (Fig. 3), after 24 h, RNAi of *CpCHSA* led to a 58.3% decrease (*P* < 0.0001, unpaired Student’s t-test) in CpCHSA at the protein level (Fig. 3a) and a 71.5% decrease (*P* < 0.0001, unpaired Student’s t-test) in *CpCHSA* mRNA level (Fig. 3b). Among the 50 pupae injected with siCHSA, 90% (*P* < 0.0001, unpaired Student’s t-test) died during the molting process from the pupal stage to the adult stage (Fig. 3c) and 75% (*p* = 0.00103, unpaired Student’s t-test) of the surviving adult mosquitoes exhibited prominent deformities (Fig. 3d).

### CpCHSA protein expression pattern

**Fig. 4:**
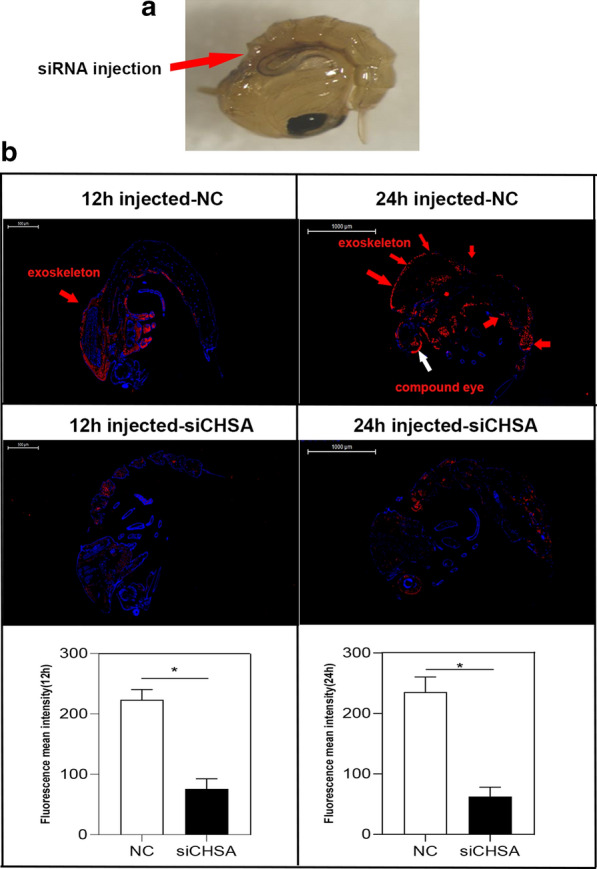
CpCHSA protein localization. Immunofluorescence analysis was performed to determine the location of CpCHSA protein expression in the pupal stages. **a** RNAi of *CpCHSA* in the pupal stage. **b** Cryosections of pupae that had been injected with siCHSA or the NC were incubated with the anti-CpCHSA antibody (red). The fluorescence was quantified using ImageJ software and expressed as the mean ± SEM (*n* = 5). Asterisk indicates a significant difference at *P < 0.05, according to Student’s t-test

We examined the pattern of CpCHSA protein expression. Immunohistochemical analysis of paraffin-embedded samples was performed using 12- and 24-h pupae (Fig. 4a). In the pupal stage, the CpCHSA protein in the NC mosquitoes was detected in the eyes and exoskeleton; however, there was almost no expression in the siCHSA group (Fig. 4b). RNAi of *CpCHSA* led to a 68.2% decrease (*P* = 0.0126; unpaired Student’s t-test) in fluorescence intensity in 12-h pupae and a 74.5% decrease (*P* = 0.0142, unpaired Student’s t-test) in 24-h pupae .

### Effect of *CpCHSA* on chitin metabolism

**Fig. 5:**
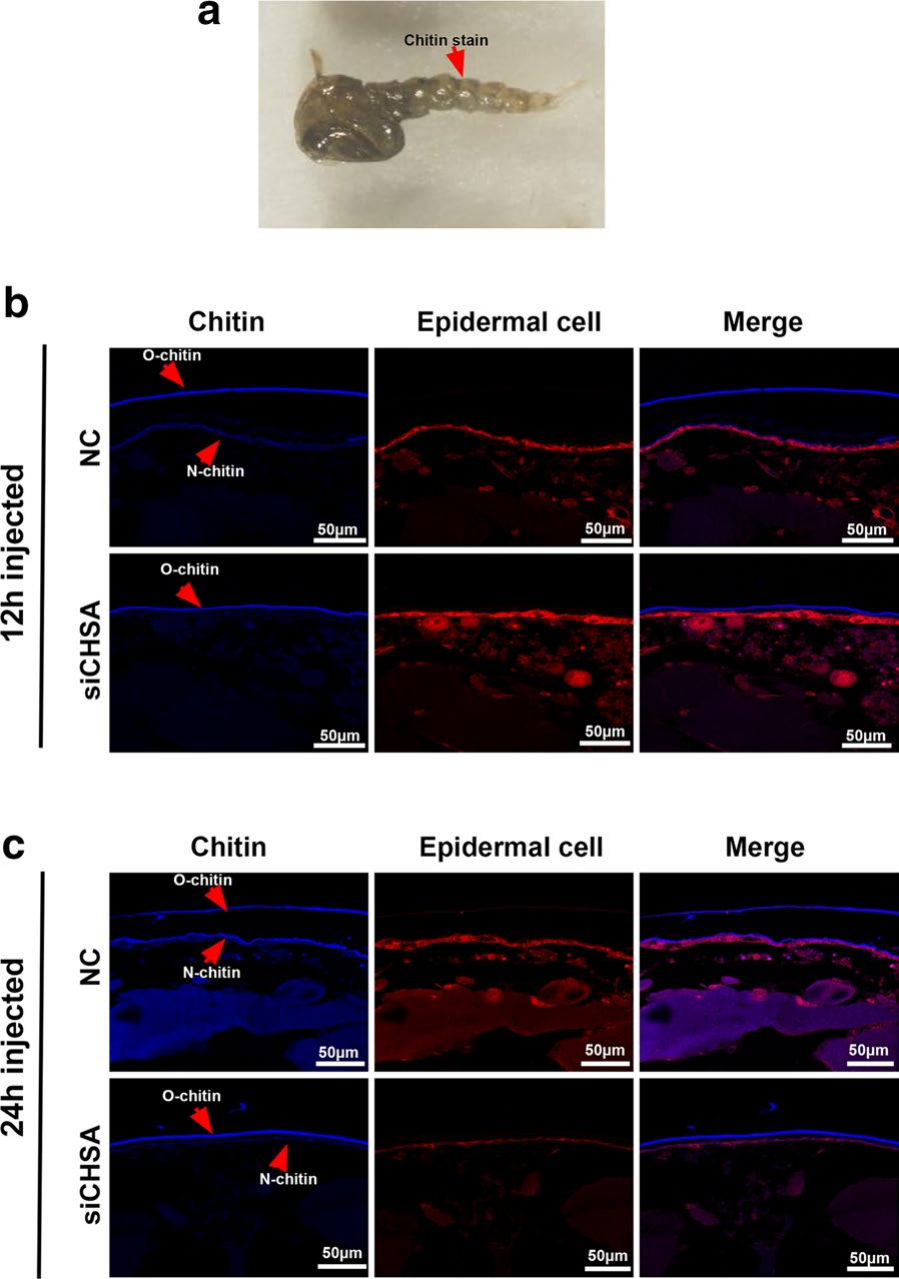
Effect of *CpCHSA* on chitin metabolism of the new and old chitin. **a** RNAi of *CpCHSA* in the pupal stage (*n* ≥ 50) The red arrow indicates the position of the collected samples. The chitin staining experiment in the integument was performed by injecting siCHSA or NC into the pupae after 12 h (**b**) and 24 h (**c**). *N-chitin* New chitin, *O-chitin* old chitin

**Fig. 6:**
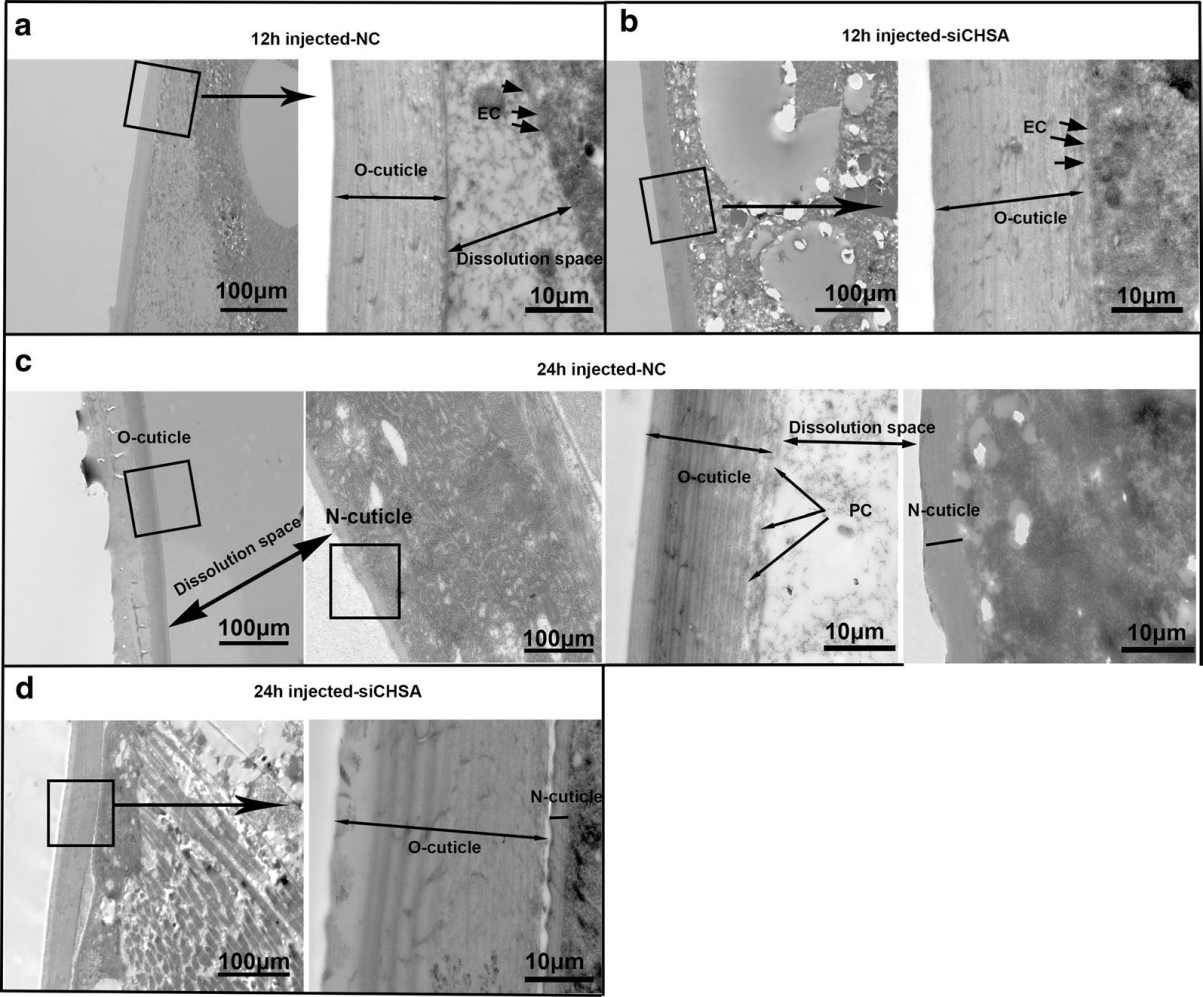
Ultrastructure of pupal cuticle from CpCHSA-deficient mosquitoes, as determined by transmission electron microscopy (TEM).** a**,** b **The pupal abdomen cuticles in the NC (**a**) and the siCHSA groups (**b**) at 12 h after injection.** c**,** d **The pupal abdomen cuticles in the NC (**c**), and the siCHSA groups (**d**) at 24 h after injection. *EC* Epithelial cell, *PC* pore canal

To further determine the effect of knockdown of *CpCHSA* expression on chitin content and the cuticle, we injected siRNA into 0- to 1-h pupae and then performed chitin staining (Fig. 5) and TEM (Fig. 6) on the abdominal integument at 12 and 24 h after injection (Fig. 5a). At 12 and 24 h after injection, the old cuticle and chitin were separated from the epithelial cell layer in the NC group, while in the siCHSA group it was not. These results show that after 12 h, siCHSA hindered the separation of old chitin from the epithelial cell layer (Fig. 5b) and prevented the separation of the old cuticle from the epithelial cell layer (Fig. 6a, b). At 24 h after injection, we observed that new cuticle and chitin were formed and that the structure of the old cuticle and chitin was incomplete in the NC group. In the siCHSA group, the formation of new cuticle and chitin was inhibited (Fig. 5c), and the structure of old cuticle and chitin was complete (Fig. 6c, d). These results show that knockdown of *CpCHSA* transcription inhibited the degradation of the old cuticle and the formation of new cuticle, which was caused by diminished chitin synthesis and degradation. RNAi resulted in the rigid structure of the old cuticle being more complete than that of the NC group, which would obstruct shedding of the old cuticle during molting.

### *CpCHSA* is required for the cuticle

**Fig. 7:**
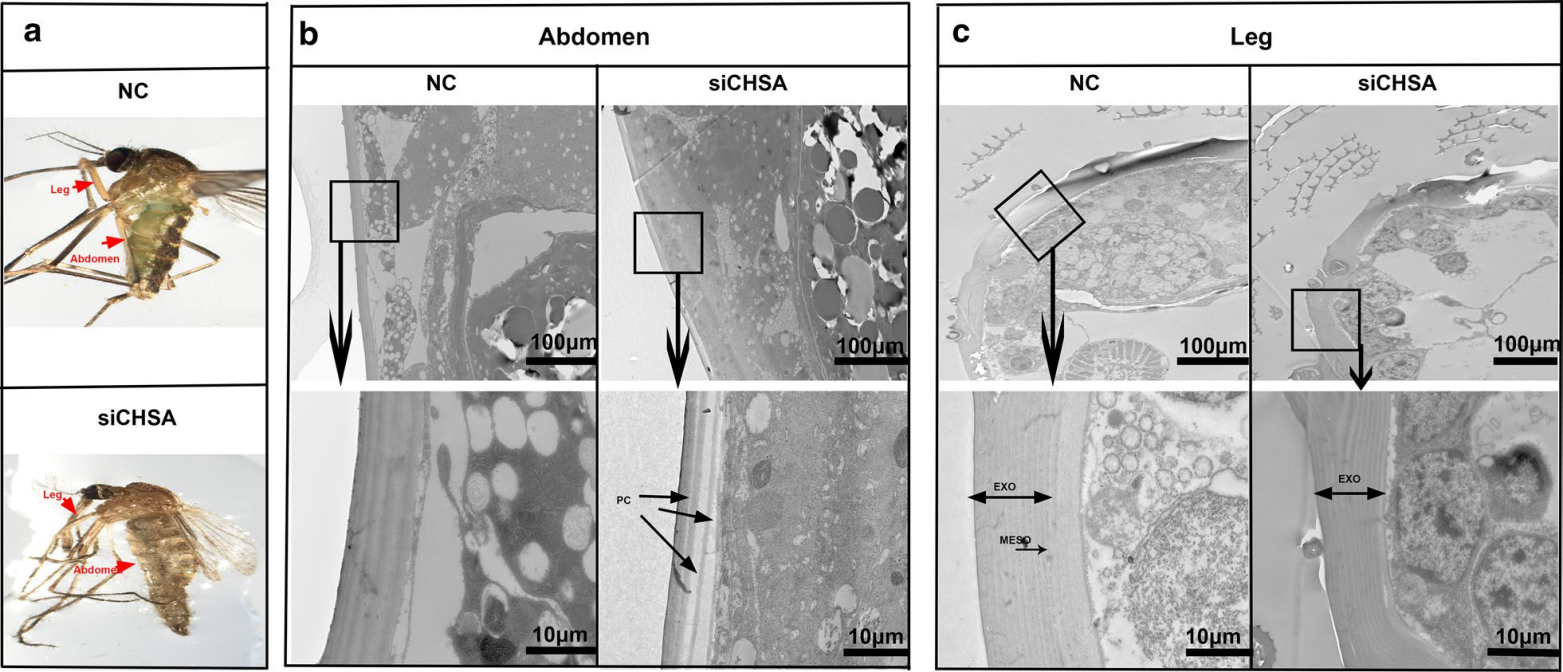
Ultrastructure of leg and abdominal cuticles from CpCHSA-deficient adults. NC or siCHSA was injected into 0- to 1-h pupae (300 ng per pupae). **a** The ultrastructure of adults at 24 h PE was analyzed using TEM. Insects were injected with NC or siCHSA. The red arrow indicates the leg and abdomen of the adult. b–c Between the NC and siCHSA groups, there were differences in the thickness and structure of the abdominal cuticle (**b**) and differences in the leg cuticle (**c**). *EXO* Exocuticle, *MESO* mesocuticle

The cuticle of insects consists of the envelope, epicuticle and procuticle [44]. The procuticle can be further divided into the exocuticle, endocuticle and mesocuticle, with horizontally aligned chitin–protein-rich laminae [45, 46]. We observed the effect of siCHSA on the ultrastructure of the cuticle using TEM (Fig. 7). Injection of siCHSA into L3 larval and 0- to 1-h pupal mosquitoes did not prevent all pupae from molting. The structure of the cuticle in the abdomen of RNAi mosquitoes had looser and less compact laminae compared with that of the control at 1-day PE (Fig. 7b). In addition, knockdown of *CpCHSA* resulted in an irregular leg structure and indistinct boundaries in the procuticle, whereas the leg of NC exhibited a normally organized, complete structure with uniform thickness (Fig. 7c).

### *CpCHSA-*deficiency results in abnormal adult cuticle

**Fig. 8:**
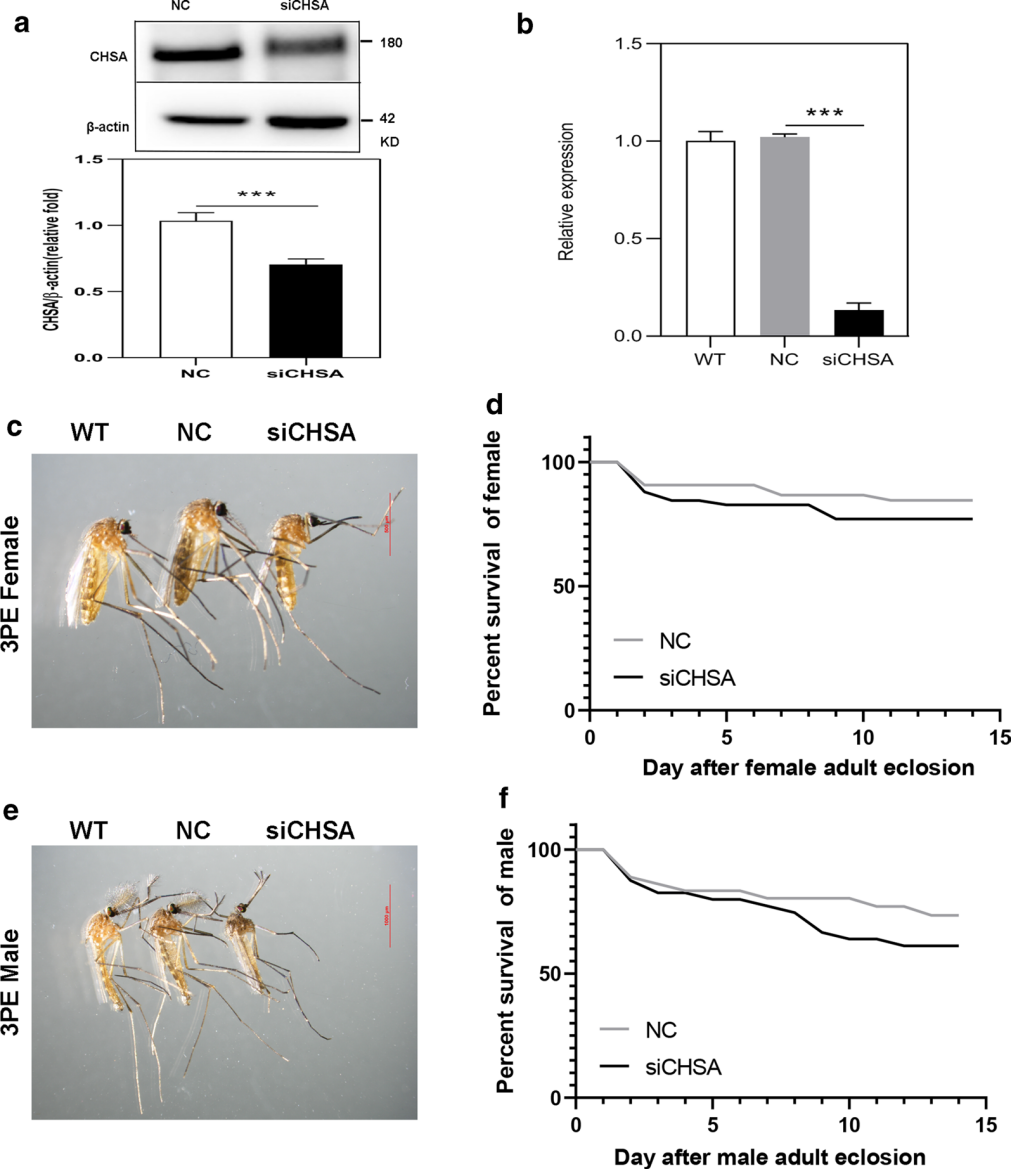
Phenotypes produced by RNAi of *CpCHSA* in the adult stage. The 12- to 24-h PE mosquitoes were injected with siCHSA (300 ng per mosquito) or NC. **a** At the 72-h PE stage, CpCHSA protein levels were determined using western blotting analysis with a CpCHSA-specific polyclonal antibody. **b** qPCR analysis of *CpCHSA* gene expression level at the 72-h PE stage. **c**, **e** Injection of siCHSA had no effect on the adult phenotype. **d**, **f** Kaplan–Meier survival curves were used to determine the female and male adult survival rates. The results are shown as the mean ± SEM. Asterisks indicate a significant difference at ***P < 0.001, according to Student’s t-test

**Fig. 9:**
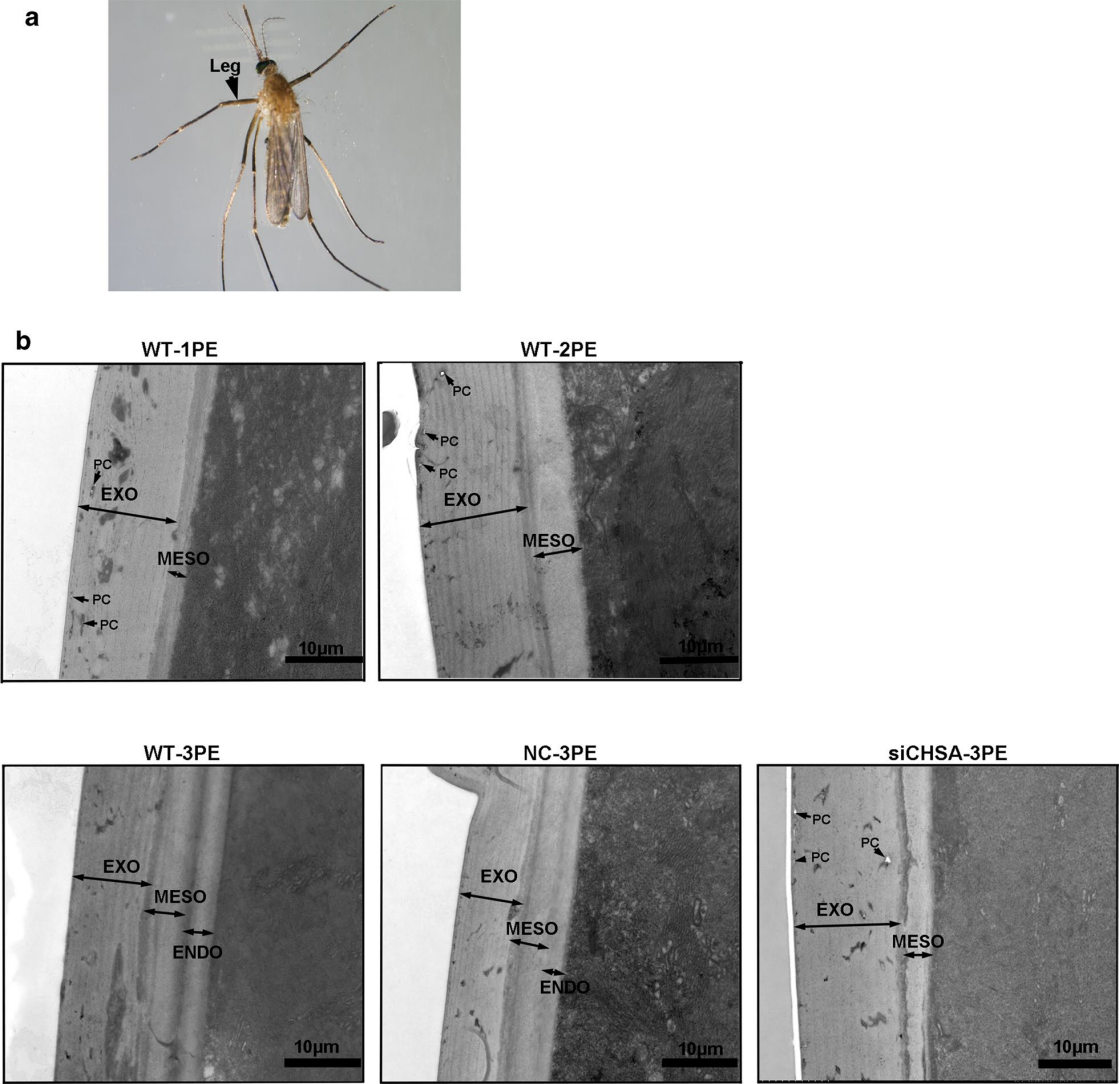
Analysis of the ultrastructure of mosquito leg cuticles. **a** The 12- to 24-h PE mosquitoes were injected with siCHSA (300 ng per mosquito) or NC (controls). After 72 h, legs were collected from the PE mosquitoes. The black arrow indicates the location at which the sample was collected. **b** Representative TEM images of leg cuticles from WT, NC and siCHSAL PE mosquitoes at 24, 48 and 72 h. *ENDO* Endocuticle

To further assess the function of *CpCHSA* in the adult mosquito, we injected siCHSA into 1-day PE mosquitoes. RNAi of *CpCHSA* led to a substantial decrease of 32.1% (*P* < 0.0001, unpaired Student’s t-test) in CpCHSA protein level (Fig. 8a) and a decrease of 89.7%, (*P* < 0.0001, unpaired Student’s t-test) in the expression of the *CpCHSA* mRNA (Fig. 8b). However, no significant morphological abnormalities (Fig. 8c, e) nor a different survival rate (Fig. 8d, f) were observed in either the siCHSA or NC group. In contrast, at 3 days PE, the ultrastructure of the endocuticle of the leg from siCHSA-treated insects was abnormal (Fig. 9a). As reported in several other insects, two distinct layers of chitin have been reported in the epidermis after adult molting. One, called the “mesocuticle”, forms underneath the exocuticle after adult eclosion, and the other, called the “endocuticle”, continues to be deposited below the mesocuticle, as shown in Fig. 9b for wild-type mosquitoes. To analyze whether *CpCHSA* deficiency affects the ultrastructure of the mesocuticle and endocuticle, the leg cuticle microstructure of adults aged 1 and 3 days was analyzed. At 3 days after eclosion, the cuticle of the leg of the WT insect contained a mesocuticle and endocuticle with a normal appearance; in contrast, the mosquitoes treated with siCHSA had only mesoderm at the corresponding stage, with no apparent endoderm structure (Fig. 9).

## Discussion

Research on the relevance of chitin to molting has been documented in many insect species; however, related information in mosquitoes is limited [6, 26, 33]. CHSA is a key enzyme in the synthesis of chitin, which is important for the development and growth of insects [21]. In the present study, knockdown *CpCHSA* in *C. pipiens pallens* resulted in failure of the molting process and failure of the cuticle structural integrity in adult mosquitoes, which suggested that CHSA is essential for the growth and development of mosquitoes. *CpCHSA* is expressed in the cuticle during molting, which has been well documented in insects [23]. In our study, *CpCHSA* expression was not limited to the molting process and cuticle, but was detected at all developmental stages and in all tissues (Fig. 1). The expression of *CpCHSA* was highest in the pupal stage, followed by that in the adult mosquito. Further analysis showed that the expression of *CpCHSA* was higher mainly in the exoskeletons of larvae and in non-blood meal-fed female mosquitoes. The qPCR results demonstrated that *CpCHSA* was also highly expressed at 24 h after egg deposition (Fig. 1a), which might imply its roles in the embryonic development of *C. pipiens pallens* eggs [26]. Moreover, in our research center, experiments showed that the expression of *CpCHSA* increased after blood-feeding by female mosquitoes, which might be necessary for ovarian development. Some studies have shown that a chitin-like material is a component of *Aedes aegypti* eggs [25]. We believe that *CPCHSA* might play an important role in the development of the egg shell, but this needs to be confirmed in subsequent experiments. The tissue expression data showed that *CpCHSA* was also highly expressed in the hindgut of the larval stage and in the foregut of the adult stage (Fig. 1a, b). The hindgut is responsible for the excretion of food residues, renal excretion, [47] and reabsorption of water and ions in most insects [48]. The main function of the foregut is to grind and temporarily store food [49]. In the larval stage, the larvae were fed grated rat chow and lived in water, which requires digestion and excretion through the hindgut, and in the adult stage, mosquitoes need to feed on sugar water or blood. The expression of *CpCHSA* in the foregut and hindgut in different developmental stages might be related to the environment and food sources. Furthermore, we detected CpCHSA protein expression in the pupal stage (Fig. 4). Immunohistochemistry showed that CpCHSA was mainly expressed in the eyes and exoskeleton. After 24 h of pupal development, CpCHSA was detected in the pupal eye. We believe that CHSA is related to eye development in the pupal and adult mosquito and that it plays an important role in the vision of mosquitoes. In *Anopheles gambiae*, chitin synthases proteins were detected in compound eyes of pupae [26]. Taken together, these results prompted us to hypothesize that, in addition to a role in the cuticle during molting, CpCHSA might be required to ensure normal growth and development of the body and physiological functions in mosquitoes.

RNA interference was applied at different developmental stages of *C. pipiens pallens* to study the function of CpCHSA systematically. Lethal phenotypes were observed in mosquitoes treated with RNAi for *CpCHSA* at most developmental stages. *CpCHSA* appears to be indispensable in the process of molting. Treatment with siCHSA led to the death of mosquitoes at the time of molting, and the death rate was highest in the pupal–adult stage, in which almost all pupae died as pharate adults entrapped in the old pupal cuticle; defects in surviving adult mosquitoes were also observed (Figs. 2, 3). Defective adult mosquito exhibited wrinkled wings and thinner abdomens, as well as curved legs that could not support flight, feeding and standing; these defective mosquitoes died within 1–7 days (Fig. 3d). These results suggest that CpCHSA plays an essential role in molting [44]. However, using chitin staining, we observed a reduction of the chitin content in the new cuticle and increased chitin in the old cuticle of the abdomen cuticle after RNAi of *CpCHSA* compared with that in the controls. The old chitin could not be separated from the epidermis after RNAi of *CpCHSA*, and the old chitin was thicker after injection of siCHSA compared with the controls (Fig. 5). A TEM study of the pupal abdominal cuticle also showed the same phenomena (Fig. 6). The cuticle consists of many thin layers with alternating dense layers. In the siCHSA group, the old cuticle was compact and intact. It extended directly from the apical membrane of the basal epidermal cells to the epidermis. In contrast, the old cuticle ultrastructure was disrupted in the NC group of insects. The inner and intermediate areas of the cuticle showed thin, unorganized sections with a low degree of compactness. The siCHSA group showed a thinner new cuticle, and the new and old epidermis could not be separated. Chitin degradation disorders result in dense and hard old epidermis, which inhibits shedding during molting. During development, insects must periodically molt to accommodate growth and overcome the rigid constraints imposed by the chitin exoskeleton [30]. The molting process begins when the epidermis secretes the outer layer of the new cuticle, separating the epidermis from the overlying old cuticle. A “dissolution space” is then formed to separate the new (internal) cuticle from the old (external) cuticle [11]. The old cuticle is qualitatively digested by chitinases, and then falls off to promote the molting process as the exuvia during a molt; the chitin in the new cuticle is promoted by chitin synthetase-mediated synthesis, which facilitates insect survival [39]. The results of our study suggest that CpCHSA is essential for the degradation of the old chitin and old epidermis and the formation of the new chitin and new epidermis during molting. This unexpected finding suggests that CHSA is not only involved in the process of molting, but may also affect chitin degradation. Chitin synthases are involved in chitin synthesis, and chitinases are involved in chitin degradation, which are two different processes. One possibility is that CHSA can affect the degradation of chitin; to assess this possibility, in earlier studies, we tested the expression of molting-related chitinases (CHT2, CHT5, CHT6, CHT8, CHT10, CHT12) after interference with *CpCHSA* [29, 30, 39]. NC and siCHSA were injected into mosquitoes in the pupal stage and the mosquitoes were collected 24 h after injection to detect the expression of chitinase. Compared with the NC, CHT5, CHT6, CHT8 and CHT13 levels were decreased after interference with *CpCHSA*, CHT2 and CHT10 levels were increased in the articular surfaces and there was no significant difference in CHT12 (data not shown). These results suggest that RNAi of CpCHSA might affect the expression of some chitinases. The chitinase family has many members, with different members possibly playing different roles in different developmental phases or in different tissues. Therefore, whether the degradation of old membranes by chitinase is associated with the active synthase synthesizing the new cuticle requires further research. RNAi for *CpCHSA* did not result in 100% molting failure; approximately 10% of the treated adults survived, although with malformed wings, legs and abdomens. We also observed a reduction in the thickness and a loss of organization of both the abdomen and leg after RNAi for *CpCHSA* compared with these properties in the controls, which would affect cuticle rigidity (Fig. 7). In the adult mosquito treated with siCHSA, the cuticle lacked an ordered layered or thick fiber structure at the corresponding stage. The cuticle has an abnormal shape, and the fiber structure is disordered and amorphous. All of these results indicate that CpCHSA is essential for the formation of proper lamellar tissue in the adult cuticle. Taken together, CpCHSA is essential for the molting process. Therefore, it would appear to be possible to control the adult mosquito population and reduce the spread of disease by inhibiting the expression of CHSA in the larval or pupal stages.

The application of siCHSA in the larval and pupal stages significantly reduced the thickness and density of the cuticle layer in the abdomen and legs of adult mosquitoes compared with those in the control group. The qPCR results indicated that *CpCHSA* was highly expressed in the wings and legs of adult mosquitoes (Fig. 1c). Therefore, we investigated the function of CpCHSA in the adult stage. Silencing *CpCHSA* did not result in mortality and deformity during adult development (Fig. 8). One possible explanation is that the laboratory environment represents an ideal environment, in which there is sufficient food and suitable conditions, resulting in low survival pressure on mosquitoes, so there is no obvious survival difference.

We hypothesized that knockdown *CpCHSA* would cause changes in the microstructures of the adult cuticle. As reported in several other species of beetles [44], two distinct layers of chitin have been reported in the cuticle after adult molting. One is called the “mesocuticle”, which forms beneath the exocuticle 1 day after adult ecdysis, and the other is called the “endocuticle”, which continues to deposit beneath the mesocuticle 2 days after adult ecdysis. To analyze the structure and composition of the cuticle of adult mosquitoes, we observed the leg anatomy of 1- and 3-day-old adult WT mosquitoes by TEM. The cuticle of the leg of the 3-day PE mosquito could be seen to have a distinct mesocuticle and endocuticle. The exocuticle was composed of a large number of dense fibers with almost no pores. In contrast, the siCHSA-treated insects lacked the endocuticle structure, and the exocuticle contained a relatively higher number ofe pore canals, such that the cuticle could not form a dense layered structure (Fig. 9). The thickness and structure of the epidermis have been reported to be related to adult development. An intact cuticle structure helps the insect resist external pressure [50, 51]. These results indicate that CpCHSA is essential for the formation of proper lamellar organization and pore canal (PC) in the outer chitinous exocuticle, mesocuticle and endocuticle of the adult cuticle. The results of the present study show that CpCHSA is essential for the formation of intact and dense cuticle structures of adult mosquitoes.

## Conclusion

Defects in CpCHSA lead not only to fatal developmental malformation, but also affect cuticle development in the adult mosquito stage. Here, we have provided a description of the effect of CpCHSA in the larvae, pupae and adults of *C. pipiens pallens*, with the results of our study suggesting that CHSA has a broader effect on insects than previously thought. Based on these and previous results, we hypothesize that CHSA could be used as a new target in strategies designed to kill mosquitoes more efficiently and safely throughout their growth cycle. If successful, these approaches could eventually reduce the spread of mosquito-borne viral infections.

## Supplementary Information

**Additional file 1: Table S1**. Gene-specific primers for the amplification of the *CHSA* cDNA from *Culex pipiens pallens*. **Table S2.** Gene-specific primers for qPCR. qPCR, quantitative real-time PCR. **Table S3.** Gene-specific primers for RNAi. RNAi, RNA interference; siRNA, small interfering RNA. **Table S4.** *CpCHSA* sequences used for polyclonal antibody preparation.

**Additional file 2**: **Figure S1** Nucleotide and deduced amino acid sequences of *CpCHSA* from *Culex pipiens pallens* (MH013352). The stop codon (TAA) is indicated by an asterisk (*) and marked in black. The putative polyadenylation signal (AATAA) is marked in black. The amino acid sequence of the putative catalytic domain is shown in white with a black background based on a previous study [12]. The signature motifs (EDR and QRRRW) in red with a black background, and the putative N-glycosylation sites are underlined. The predicted, hydrophobic, membrane-spanning regions of the deduced amino acid sequence are shown in black with a gray background.

## Data Availability

All data are fully available without restriction.

## Abbreviations

AD: Adult
CHSA: Chitin synthase A
CHSB: Chitin synthase B
L3: Third-instar larvae
L4: Fourth-instar larvae
NC: Negative control
PBM: Post-blood meal
PE: Post-eclosion
qPCR: Quantitative real-time PCR
RNAi: RNA interference
SEM: Standard error of the mean
siRNA: Small interfering RNA
TEM: Transmission electron microscopy
WT: Wild type
