# Supplementary material for: Physiological characterization of chitin synthase A responsible for the biosynthesis of cuticle chitin in Culex pipiens pallens (Diptera: Culicidae)
Source: Parasit Vectors. 2021 May 1;14:234. doi: 10.1186/s13071-021-04741-2 (PMC8088658; doi:10.1186/s13071-021-04741-2)
Supplement: Supplementary file 1 — Additional file 1: Table S1. Gene-specific primers for the amplification of the CHSA cDNA from Culex pipiens pallens. Table S2. Gene-specific primers for qPCR. qPCR, quantitative real-time PCR. Table S3. Gene-specific primers for RNAi. RNAi, RNA interference; siRNA, small interfering RNA. Table S4. CpCHSA sequences used for polyclonal antibody preparation. [file 13071_2021_4741_MOESM1_ESM.doc]

| **Table S1. Gene-specific primers for the amplification of cDNA of CHSA from *Culex pipiens pallens*** | | | |
| --- | --- | --- | --- |
| **DNA fragment** | **Primer name** | **Primer sequence (5′→3′)** | **PCR product size (bp)** |
| 1 | CpCHSA-F1 | TCTGCTATCAGACATCGACCTC | 773 |
|  | CpCHSA-R1 | ATCAGCCACAGGATTGGTCT |  |
| 2 | CpCHSA-F2 | GATGGAATCGTTCCATACGAT | 1013 |
|  | CpCHSA-R2 | CCATCGACTGATCGATCAGA |  |
| 3 | CpCHSA-F3 | GCTCTGGTTGCTGTCTCAAA | 1059 |
|  | CpCHSA-R3 | ATTACGTTGTCGTCCATCAGA |  |
| 4 | CpCHSA-F4 | GATGGTGTGGTACCAGAAGTTC | 1139 |
|  | CpCHSA-R4 | GGTTGTCCATCTTCTTCTGGA |  |
| 5 | CpCHSA-F5 | CTGTCTGTTGTGCACGCAT | 1213 |
|  | CpCHSA-R5 | TGCTGTTGCTCCAGGTGA |  |
| 6 | 5′RACE-GSP | GATTACGCCAAGCTTCCGGTGTCCTCCTTGATCGGAGGGTC |  |
| 7 | 3′RACE-GSP | GATTACGCCAAGCTTGTGAGCGTGAAGGACATCTTCAACGTGA |  |

**Table S2. Gene-specific primers for qPCR**

| **Application of primers** | **Primer name** | **Primer sequence (5′→3′)** | **PCR product size (bp)** |
| --- | --- | --- | --- |
| qPCR | CpCHSA-F | GCTACTTCACCTACATGTTCCTATCG | 262 |
|  | CpCHSA-R | CAGCACGTAGGTAACAGTATTGTACG |  |
|  | B-actin-F | AGCGTGAACTGACGGCTCTTG | 140 |
|  | B-actin-R | ACTCGTCGTACTCCTGCTTGG |  |

qPCR, quantitative real-time PCR.

| **Table S3. Gene-specific primers for RNAi** | | |
| --- | --- | --- |
| **Application of primers** | **Primer name** | **Primer sequence (5′→3′)** |
| RNAi-Chitin Synthase A | siCHSA-F | CCGUUCGGAUAUGUUUCUUTT |
|  | siCHSA-R | AAGAAACAUAUCCGAACGGTT |
| RNAi-Negative Control | NC-F | GCGACGAUCUGCCUAAGAUdTdT |
|  | NC-R | AUCUUAGGCAGAUCGUCGCdTdT |

RNAi, RNA interference; siRNA, small interference RNA.

**Table S4. CpC**HSA sequence used for polyclonal antibody preparation

| **Sequence** |
| --- |
| CATLWHETKEEMMVFLKSIMRMDEDQCARRVAQKYLRIVDPDYYEFETHIFFDDAFEISDHSDDDIQCNRFVKILIDTIDEAASEVHQTNIRLRPPKKYPTPYGGRLVWTLPGKTKLISHLKDKDRIRHRKRWSQVMYMYYLLGHRLMELPISVDRKDVMAENTYLLTLDGDIDFNPSAVTLLVDLMKKNKNLGAACGRIHPIGSGPMVWYQKFEYAIGHWLQKATEHMIGCVLCSPGCFSLFRGKGLMDDNVMRKYTTRSDEARHYVQYDQGEDRWLCTLLLQRGYRVEYSAASDAYTHCPEGFNEFYNQRRRWVPSTIAN |
