# Supplementary figures and images for: Physiological characterization of chitin synthase A responsible for the biosynthesis of cuticle chitin in Culex pipiens pallens (Diptera: Culicidae)
Source: Parasit Vectors. 2021 May 1;14:234. doi: 10.1186/s13071-021-04741-2 (PMC8088658; doi:10.1186/s13071-021-04741-2)

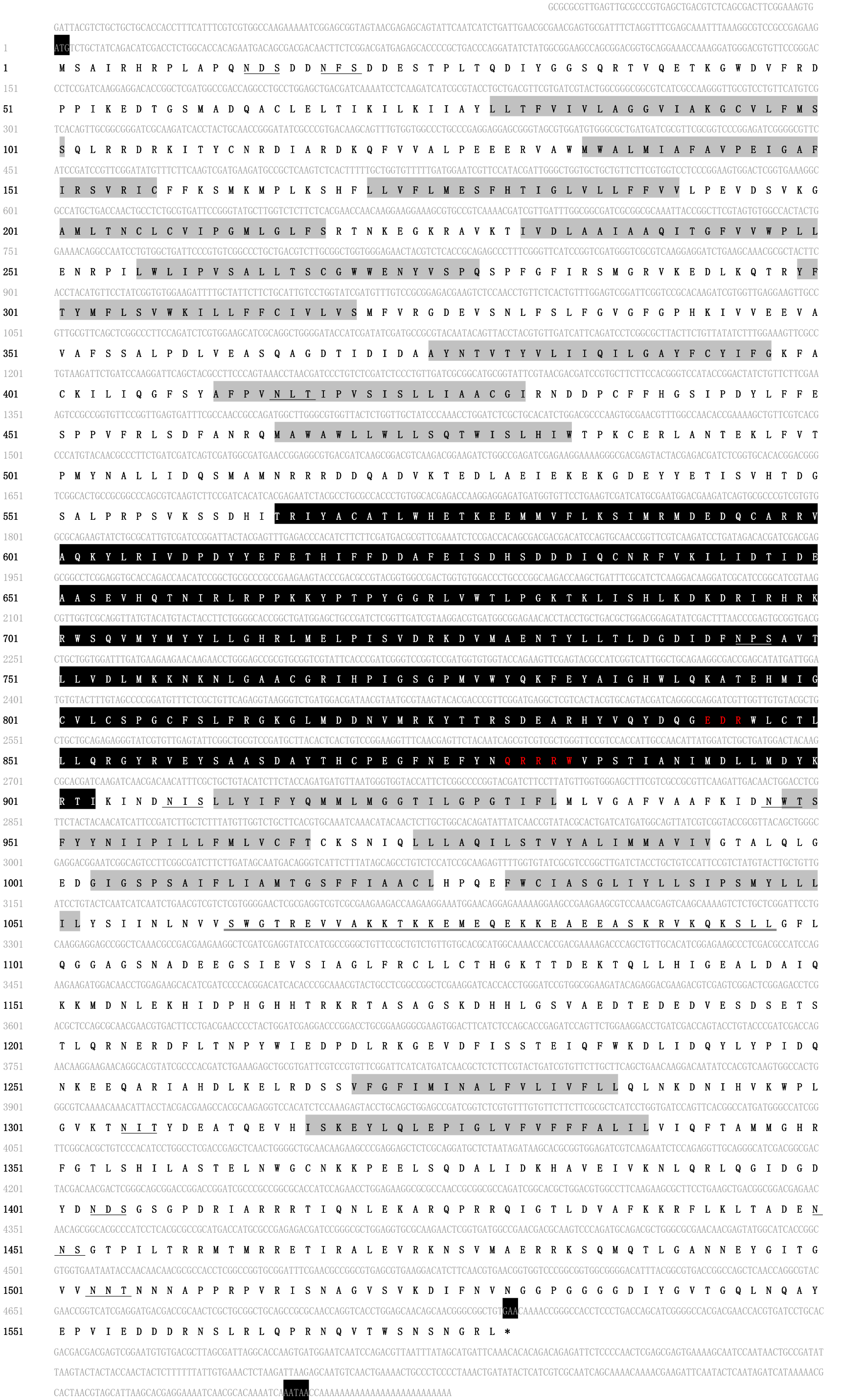

Supplement: Supplementary file 2 — Additional file 2: Figure S1 Nucleotide and deduced amino acid sequences of CpCHSA from Culex pipiens pallens (MH013352). The stop codon (TAA) is indicated by an asterisk (*) and marked in black. The putative polyadenylation signal (AATAA) is marked in black. The amino acid sequence of the putative catalytic domain is shown in white with a black background based on a previous study [12]. The signature motifs (EDR and QRRRW) in red with a black background, and the putative N-glycosylation sites are underlined. The predicted, hydrophobic, membrane-spanning regions of the deduced amino acid sequence are shown in black with a gray background. [file 13071_2021_4741_MOESM2_ESM.tif]
